# The Role of Artificial Intelligence and Machine Learning in the Assessment, Diagnosis, and Prediction of Cerebral Small Vessel Disease

**DOI:** 10.7759/cureus.93376

**Published:** 2025-09-27

**Authors:** Ali Al Askar, Divya Buchireddygari, Bose Venkata Sai Ridhira Middi, Shivram Ravishankar, Iosif Namidis, Milko Garcés, Lulu S Chamayi, Ee Tienne Ong, Muhammad Abdul-Muizz, Renata A Dias, Vishal Babu, Ramsha Ali

**Affiliations:** 1 College of Medicine, Imam Abdulrahman Bin Faisal University, Dammam, SAU; 2 Radiology, Yashoda Hospital, Hyderabad, IND; 3 Medicine and Surgery, Sri Venkateshwaraa Medical College, Tirupati, IND; 4 Medicine and Surgery, University of Central Lancashire, Preston, GBR; 5 Medicine and Surgery, University of Thessaloniki, Thessaloniki, GRC; 6 Medicine and Surgery, Universidad Peruana de Ciencias Aplicadas, Lima, PER; 7 Medicine and Surgery, Azeezia Medical College, Kollam, IND; 8 Geriatrics, Blackpool Victoria Hospital, Blackpool, GBR; 9 Medicine and Surgery, Imperial College London, London, GBR; 10 Medicine and Surgery, Amala Institute of Medical Sciences, Thrissur, IND; 11 Medicine and Surgery, New Cross Hospital, Royal Wolverhampton NHS Trust, Wolverhampton, GBR; 12 Medicine and Surgery, Peoples University of Medical and Health Sciences, Nawabshah, PAK

**Keywords:** artificial intelligence, cerebral small vessel disease, convolutional neural networks, deep learning, diagnosis, machine learning, neuroimaging, stroke, white matter hyperintensities

## Abstract

Cerebral small vessel disease (CSVD) contributes substantially to ischemic stroke and vascular cognitive impairment but remains difficult to detect with conventional diagnostics. Recent advances in artificial intelligence (AI), including machine learning (ML) and deep learning (DL), have improved neuroimaging analysis, early risk stratification, and clinical decision support in CSVD-related stroke, while raising questions about generalizability, interpretability, and ethics.

This review aims to narratively synthesize how AI supports neuroimaging analysis, early detection, clinical decision-making, and prognostication in stroke with an emphasis on CSVD, and to summarize limitations, bias, and implementation challenges.

This narrative review synthesized evidence from 122 studies. AI showed strong performance across stroke care with an emphasis on CSVD: intracerebral hemorrhage (ICH) detection on noncontrast CT (sensitivity = 93%, specificity = 92%); 18-25-minute reductions in door-to-needle time; superior prediction of 90-day disability versus clinician assessment (89% vs. 72%); reduced inter-rater variability for white matter hyperintensities (WMHs) segmentation; ~94% accuracy for enlarged perivascular spaces (EPVS) classification on MRI; and faster team notification and time-to-treatment, with mixed evidence for improved 90-day functional independence. However, performance was weaker in older and diabetic cohorts, underscoring limited generalizability, scarce prospective validation, and risks of bias.

AI augments stroke care across imaging-based diagnosis, risk stratification, and rehabilitation, with growing utility in CSVD. Translation into routine care requires robust external validation, bias mitigation, model interpretability, and clear governance around safety, liability, and cost.

## Introduction and background

Stroke refers to the rapid onset of clinical symptoms due to brain function impairment, lasting for more than 24 hours or leading to death, with no clear cause other than a vascular origin [1]. In 2017, stroke ranked as the second leading cause of death worldwide and the third leading cause of combined death and disability [2]. Delays in treatment worsen stroke outcomes. Each minute without reperfusion destroys about 1.9 million neurons, 14 billion synapses, and ~12 km (7.5 miles) of myelinated fibers; every hour of untreated ischemia ages the brain by roughly three and a half years [3].

Cerebral small vessel disease (CSVD) encompasses a variety of clinical, imaging, and pathological conditions resulting from different causes that impact the brain's small arteries, venules, and capillaries. It represents the pathological effects of small vessel dysfunction on brain tissue. The term "small cerebral vessels" includes small arteries (100-400 μm in diameter), arterioles (40-100 μm in diameter), capillaries, venules, and small veins. CSVD is responsible for 25% of all strokes and plays an even greater role in recurrent strokes, with an incidence as high as 50%. Furthermore, it contributes to 45% of dementia cases [4]. Timely clinical assessment, symptom recognition, and accurate imaging are vital for effectively managing cerebrovascular disease and prioritizing patients who may require urgent, life-saving interventions like revascularization [5].

During the development of artificial intelligence (AI) technology in recent years, a number of AI models have been created that can enhance patient care at every level, from prehospital diagnosis to rehabilitation. AI methods such as deep learning (DL) and convolutional neural networks (CNNs) have demonstrated significant promise in the analysis of stroke images, classification of imaging findings, and etiologic inference where applicable, and in assisting physicians in making quicker decisions that could lead to better results [6].

With the increasing burden of cerebrovascular disease, AI models have made more paramount advances in complex neurological disorders by automating complex tasks in an effective, timely manner, aiding physicians in making critical decisions in less time [7]. Uses of AI and machine learning (ML) models in cerebrovascular disease are largely focused on imaging modalities. RapidAI and Viz.ai are examples of commercial software AI tools that are used in ischemic and hemorrhagic strokes [5]. While these tools primarily target large-vessel occlusion triage, they illustrate end-to-end deployment features such as regulatory clearance, workflow integration, and monitoring, which are directly transferable to AI applications in CSVD. AI algorithms were created to analyze imaging data in stroke cases and help identify which patients would benefit from a thrombectomy or from thrombolysis, reducing the average diagnostic time by radiology teams and resulting in better patient outcomes [7]. Moreover, telestroke has been emphasized in literature as one of the domains of care where AI may improve remote stroke assessment and predict patient outcomes [8]. AI models are also used to augment human decision-making by analyzing large amounts of data and trying to identify patterns that may be overlooked or ignored by traditional statistical analysis [9]. Examples of these AI models include the “Hemorrhage After Thrombolysis” (HAT) score, the “Safe Implementation of Treatments in Stroke Symptomatic Intracerebral Hemorrhage" (SITS-SICH) score, and the “Stroke Prognostication using Age and National Institutes of Health Stroke Scale-100" (SPAN-100) [10]. Despite advances in AI applications for stroke care, significant limitations remain, particularly regarding generalizability and clinical applicability. Many studies lack diversity in patient populations and stroke subtypes, with a notable disproportion between anterior and posterior circulation occlusions [11].

This review evaluates the clinical significance and limitations of current advances in AI for stroke diagnosis and management. By examining cutting-edge approaches and their integration into clinical practice, the review illustrates both the transformative potential of AI and the critical gaps that remain to be addressed. As these technologies evolve, their application across diagnostic and therapeutic pathways holds substantial promise for improving patient outcomes and reducing the global burden of cerebrovascular disease.

## Review

Methods

We have conducted a narrative review using the literature extracted from PubMed up until January 2025. The search mostly resulted in review articles, systematic reviews, meta-analyses, randomized controlled trials (RCTs), and textbook publications. The keywords used in our search were as follows: ((AI[Title] OR artificial intelligence[Title] OR machine learning[Title]) AND (cerebrovascular[Title] OR stroke[Title])) AND (use[Title] OR diagno*[Title] OR asses*[Title] OR predic*[Title]).

Results came back with a total of 390 publications. We initially screened titles and abstracts for relevance, followed by the application of predefined inclusion and exclusion criteria, which resulted in the selection of clinically salient and methodologically informative studies. Accordingly, we included articles discussing AI or ML tools applied to clinical practice, imaging modalities, electronic devices, or biomarkers within the context of cerebrovascular disease. To ensure methodological rigor, we prioritized studies with robust designs (e.g., prospective cohorts, large sample sizes, and external validation), as well as those introducing novel algorithms with clear benchmarking against established methods. Our objective is to synthesize AI applications with a primary emphasis on CSVD hallmarks and related biomarkers, while using non-CSVD stroke tools only as brief comparators for implementation maturity.

Inclusion Criteria

Study designs: Review articles, systematic reviews, meta-analyses, RCTs, and textbooks (for definition/background purposes only), written in English.

Publication date range: Including studies from 2015 to 2025.

Topic relevance: AI use related to CSVD, including any of the following domains: imaging markers, clinical, biomarker, or genetic/omics data. Studies on cerebrovascular disease without an explicit CSVD linkage were excluded. Single-domain studies (e.g., biomarker-only) were eligible if the domain was evaluated in relation to CSVD.

Data outcome relevance: Performance metrics of ML programs used in electronic device technologies and tools that assess, diagnose, or predict cerebrovascular disease.

Exclusion Criteria

Exclusion criteria included the following: (1) non-English publications; (2) non-relevant diseases, such as Parkinson's, epilepsy, heart disease, or other non-relevant conditions; (3) irrelevant uses, such as AI use at a hospital management or administration level.

After removing duplicates, we screened titles and abstracts for relevance to CSVD and the English language. Potentially eligible articles underwent full-text assessment against predefined inclusion/exclusion criteria (e.g., non-CSVD diseases, administrative/non-clinical AI uses, non-AI focus, and non-English studies). Screening was performed independently by two reviewers; disagreements and borderline cases were adjudicated in Rayyan (Cambridge, MA) by a six-author panel via discussion and majority vote. In total, 122 studies were retained for qualitative synthesis. We then conducted a narrative analysis of their effectiveness and limitations. To mitigate publication bias, we applied predefined selection criteria, dual independent screening, and multi-reviewer adjudication. As this is a narrative synthesis across heterogeneous designs, a PRISMA (Preferred Reporting Items for Systematic Reviews and Meta-Analyses) flow diagram was not performed; instead, we emphasize conceptual breadth, clinical context, and methodological limitations.

Results

A total of 122 studies, including 11 RCTs, 23 systematic reviews, and nine meta-analyses, met the inclusion criteria to evaluate the potential and limitations of AI in cerebrovascular disease. In addition to highlighting important dangers and gaps in clinical validation, the findings show how AI might improve diagnostic accuracy, streamline workflows, and predict outcomes in situations, including acute stroke and CSVD. Across CSVD hallmarks, AI systems demonstrated improved segmentation and grading performance with potential for standardized burden assessment.

Acute Ischemic Stroke: Triage and Imaging

AI systems have successfully reduced time-sensitive delays in acute stroke therapy. In multicenter trials, DL models reported a pooled sensitivity of 93% (95% CI: 89-96%) and a specificity of 92% (88-95%) for intracerebral hemorrhage (ICH) detection on noncontrast CT [12]. Commercial AI triage platforms increased reperfusion rates in ischemic stroke by cutting door-to-needle times by 18 to 25 minutes [13]. Prehospital ML methods fared better than clinical measures like RACE for large vessel occlusion (LVO) detection, with an area under the curve (AUC) of 0.89-0.93 vs. 0.75-0.82. Prognostic accuracy was also improved by AI; integrated imaging-clinical models predicted three-month post-stroke impairment (modified Rankin Scale (mRS) ≥3) with 89% accuracy, compared to 72% for clinician assessment alone [14]. The AI sensitivity for LVO detection decreased to 82% (77-86%) in individuals over 80 years, and diabetics had lower calibration accuracy (Brier score: 0.21 vs. 0.13 in non-diabetics), according to subgroup analyses that revealed performance disparities [15]. These results demonstrate that to reduce biases in retrospective datasets, population-specific algorithm training is required. Most models were trained on single-center cohorts with limited external validation, which may inflate performance at deployment.

CSVD: Imaging Biomarkers and Prognosis

AI models have been used to predict outcomes in CSVD (e.g., lacunar stroke) and to estimate risk using biomarkers (e.g., neutrophil activation markers) [16]. ML techniques have demonstrated significant potential in the identification, assessment, and prediction of CSVD. AI models, such as CNN-based models, emerge as powerful tools to overcome the inherent limitations of traditional diagnostic methods like MRI, which rely on human interpretation and are prone to biases and subjectivity [17]. AI-based algorithms have been employed to automate the segmentation and classification of white matter hyperintensities (WMHs) and to stratify the severity of CSVD. For instance, Lambert et al. [18] used an automated algorithm to assess the severity of WMHs, while González-Castro et al. [19] developed an automatic scheme to classify enlarged perivascular spaces (EPVS) on MRI, reporting 94% accuracy (95% when compared to human approaches). CNN achieved a Dice score of 0.88-0.92 for WMHs segmentation [20]. ML models using neutrophil activation biomarkers predicted lacunar stroke recurrence risk (HR: 1.28; 95% CI: 1.12-1.46) [19]. Although these developments encourage earlier intervention in CSVD, validation across a range of populations is still required because geriatric and multiethnic groups are underrepresented in training datasets [21,22]. However, geriatric and multi-ethnic cohorts remain under-represented, limiting generalizability.

Prognostication and Treatment Selection

AI's clinical utility in treatment selection is further highlighted by its incorporation into new treatments. ML models (AUC: 0.91; 95% CI: 0.87-0.94) outperformed clinical scores (AUC: 0.76) for predicting post-thrombolysis hemorrhagic transformation [23]. In a pre/post implementation study of an AI triage platform for LVO, median time to neuroendovascular team notification decreased (25.0 vs. 40.0 minutes; p = 0.01), while 90-day functional independence (mRS ≤ 2) was numerically higher (33.3% vs. 22.2%) but not statistically significant (p = 0.52) [24].

Bias, Generalizability, and Validation Gaps

The diagnostic accuracy of AI models is heavily influenced by the quality and reliability of training data annotations, significantly so in medical imaging. Two key issues are the "black-box" nature of some models and dataset shift/spectrum bias from historical training data, which can inflate apparent performance. Across studies, performance tended to dip in earlier-stage disease and in resource-limited settings, with more false-positive alerts reported in some deployments; small, single-center datasets, heterogeneous pipelines, and limited external validation further constrain generalizability [25]. Concerns regarding the generalizability of AI models are raised by the fact that only 33% of them have been verified in populations outside of stroke centers [26]. Prospective multicenter trials, explainable frameworks, and calibrated reporting are needed for reliable deployment.

Discussion

AI in CSVD-Related Stroke

CSVD is an umbrella term for a wide array of underlying causes and mechanisms affecting the perforating cerebral arterioles, venules, and capillaries. Presentations can range anywhere from being completely asymptomatic to focal neurological deficits (strokes: hemorrhagic and ischemic presentations), to global neurological dysfunctions and dementia. In fact, about one-fifth of ischemic strokes and about 45% of dementias can be attributed to CSVD. CSVDs are also associated with an increased risk of stroke recurrence by more than 50%. Masses affected by this significant public health problem usually end with cognitive decline, motor dysfunction, and even psychiatric disorders. CSVDs are also found to deleteriously impact the post-stroke recovery period due to their interference with the reorganization of brain networks, thereby leading to inferior functional outcome [27]. They are also known to trigger a cascade of events that involves the extension of the lesion to secondary locations [28]. CSVD is associated with increased risk of stroke-associated pneumonia after intracerebral hemorrhage, irrespective of the volume. This was identified by Zhang et al. (2024) using a logistic regression model [29]. A wide spectrum of genetic causes has been implicated in the development of CSVD, including Fabry disease and CADASIL, CARASIL, MELAS, COL4A1, and TREX1 mutations.

Polygenic risk scores (PRS) using the linkage disequilibrium-predicted (LDpred) algorithm and PRS-CS, which incorporates a Bayesian regression framework and applies continuous shrinkage to single-nucleotide polymorphism (SNP) effect sizes, rely on linear regression with summary statistics from genome-wide association studies (GWAS) and genetic meta-analyses. These approaches represent AI-based models to detect possible genetic causes underlying CSVD. The shared genetic architecture of WMHs and cardiovascular traits, including systolic blood pressure (SBP), diastolic blood pressure (DBP), lifetime smoking index, myocardial infarction (MI), and risk of venous thromboembolism (VTE), as well as the polygenic mechanisms of cardiovascular traits and CSVD, demonstrated through PRS association studies and linkage disequilibrium score regression, have contributed significantly to genetically based precision medicine. Pathway-specific PRS, using a variety of complex algorithms, help detect both known and novel etiologies, and certain PRS (e.g., from the endothelial cell apoptosis pathway), when combined with clinical features, can predict post-ischemic stroke mortality. The use of ML algorithms to integrate pathway-specific PRS with outcome prediction models may further enhance predictive performance [30].

Yim et al. (2021) [31] established that the diagnosis of Fabry disease is associated with significant challenges due to the asymptomatic nature of non-classical cases, limited access to advanced imaging in remote centers, and clinicians’ difficulty in reaching accurate diagnoses because of nonspecific findings and limited disease-specific expertise. Models predicting left ventricular thickness, 12-lead ECG, and late gadolinium enhancement (LGE) can help address these issues. Gervas-Arruga et al. (2024) [32] developed an in silico ML-based program, which, when combined with clinical features, facilitated the early detection of biomarkers indicating vascular and neural damage in Fabry disease.

Another model, which used fiber evanescent wave spectroscopy bolstered by ML, identified it to be a more economical method for screening for early diagnosis and monitoring [33]. Montella et al. (2024) [34] used a DL based model to predict the brain age and established that Fabry disease is associated with older appearing brains and brain predicted age difference as a disease severity marker. Endothelial dysfunction and loss in integrity of the blood-brain barrier (BBB) due to hemodynamic changes, including loss in the auto-regulation of cerebral blood flow with ageing and elevated pulsatility in the cerebral arterioles due to stiffening of blood vessels, is one of the most important underlying pathophysiology of CSVD. Other contributors include inflammation with leukocyte infiltration and oxidative damage due to excessive reactive oxygen species (ROS) formation.

In pursuit of suitable biomarkers to identify patients with CSVD and predict the underlying etiology, markers of endothelial dysfunction (ICAM1, VCAM1, CD62E, CD62P), markers of inflammation and neutrophil chemotaxis (myeloperoxidase (MPO) and calprotectin), and markers establishing the role of platelets (CXCL4 and 7) were included. ML models were developed to further establish the role of these in detecting and predicting CSVD. In the linear regression-based learning algorithm, the combination of MRI and patient characteristics was considered superior in predicting over the enzyme-linked immunosorbent assay (ELISA) biomarkers, and MPO and CXCL4 were considered predictors (>80% specificity; <40% sensitivity. A decision tree-based random forest model, on the other hand, established that the levels of these biomarkers are important in determining the cutoff value (MPO, low-density lipoprotein, CXCL4, MPO DNA S100A8/A9) of 100% sensitivity, defective in differentiating between controls and cohorts [35]. Another study produced a neural network model to appropriately segment deep medullary veins and establish them to be one of the pathologies underlying CSVD. Oxidative damage was a possible underlying mechanism of the damage caused in CSVD. AI can be used to detect the levels using wearable devices, classify, and predict the oxidative stress [36].

MRI is the most sensitive modality used to diagnose CSVD. The STRIVE 2 guidelines state that a combination of any of the following eight features observed on MRI is associated with the diagnosis: recent small subcortical infarcts, lacunae of presumed vascular origin, WMHs, EPVS, cerebral microbleeds (CMBs), cortical superficial siderosis, brain atrophy, and cortical cerebral microinfarct. Hu et al. (2024) [4] emphasized the fact that the use of AI, especially convoluted neural networks, in identifying these lesions is beneficial in enhancing the diagnostic accuracy and understanding of the disease. These models included efficient segmentation, followed by analysis of the images. Li et al. (2025) [37] developed DDEvENeT, which included an amalgamation of DL and ensemble strategies that proved to be superior to the existing models for segmentation and parcellation, contributing significantly to early and more efficient diagnosis. Pantoni et al. (2019) [38] established the role of cerebral white matter fractal dimension (FD) and cortical gray matter FD from high-resolution T1-weighted images using an ML model based on LASSO (least absolute shrinkage and selection operator) regression. The database used included standard and advanced neuroimaging features. White matter FD complexity decrease coincides with worsening cognitive impairment. CMBs are a diagnostic imaging marker for CSVD, which occurs because of intravascular hemosiderin deposition, and are associated with cognitive impairment, including memory and executive dysfunction. The location of CMBs can provide insight into the underlying etiology. Deep brain CMBs are typically associated with hypertension, while lobar CMBs are more suggestive of cerebral amyloid angiopathy (CAA). Therefore, it is of great prognostic and diagnostic significance if we could map the location and quantify the CMBs. A variety of ML algorithms have been developed to assist with this arduous and tedious process of manually detecting CMBs. A 50% sensitivity was achieved by Seghier using an automated segmentation method, while the radial symmetry transform method by Kuijf et al. (2012) [39] increased it to 71.2%. Further improvement in the model came in through Bian et al. (2013) [40], increasing the sensitivity to 88.4% by proposing a 2D fast radial symmetrical transformation. Manual feature extraction was added to further enhance the results by Chen et al. (2019) [41]. All these models used susceptibility weighted imaging (SWI) MRI and required manual checks. Xia et al. (2023) [42] came up with a two-stage parameter-free DL model to spontaneously identify CMBs on quantitative susceptibility mapping (QSM) (more exact quantification of CMBs and the capability to ascertain CMBs from calcifications) with a combined sensitivity of 88.9% and false positives per subject of 2.87. It also included identifying the precise location of the microbleeds as per the microbleeds anatomical rating system, with a sensitivity of 85% for nine brain regions. Wu et al. (2023) [43] emphasized the use of a masked region-based convolutional neural network (R-CNN), an end-to-end algorithm, and CMBs segmentation masks, which ensured a lesser scope for error and effectiveness in detecting and classifying CMBs based on the etiopathology (arteriolosclerosis (aSVD), cerebral amyloid angiopathy (CAA), cerebral autosomal dominant arteriopathy with subcortical infarcts and leukoencephalopathy (CADASIL)). This showed greater efficacy than the neurologist group and also eliminated the requirement for further diagnostic tests like biopsies and genetic testing to confirm the diagnosis, proving to be more cost-effective. Lu et al. (2022) [44] developed a nonlinear learning algorithm using HYDRA, a semi supervised ML program for a combination of binary classification and grouping together of sub populations and managed to classify CSVD into three types, each distinctive in terms of the variation in the cerebral blood flow (CBF) patterns and also in terms of gender and proportion of various imaging findings (Table 1), CSVD burden, and possible risk factors and clinical manifestations to ensure a more patient specific diagnosis and treatment.

**Table 1:** Summary of CSVD subtypes based on the HYDRA (Heterogeneity through Discriminative Analysis) algorithm. Source: [44]. CSVD: cerebral small vessel disease; CBF: cerebral blood flow; PVWMH: periventricular white matter hyperintensities.

Type	Type 1	Type 2	Type 3
Gender	Male > Female	Female > Male	Male > Female
CBF	Decrease: Left temporal lobe. Increase: Right parietal and occipital lobes	Decrease: Right hemisphere of the brain. Increase: Left cerebrum	Decrease: Posterior part of the brain. Increase: Anterior part of the brain
Lacunae and PVWMH	Lesser possibility	Higher possibility	Higher possibility
Risk factor controls that are most beneficial	Smoking	Blood pressure	Smoking and blood pressure
Other	-	-	CBF was negatively associated with total CSVD burden

Different cardiovascular risk factors presented with varied locations and morphologies of WMHs, as established by Keller et al. (2022) using a linear regression model [45]. Another 3D DL model was developed for grading the severity of perivascular spaces for the prognosis of CSVD by Williamson et al. [46].

Li et al. (2022) came up with an end-to-end 3D convolutional network to detect branch points in vessels [47]. Hsieh et al. (2019) developed a CNN to detect CSVD, identify the exact coordinates of the block, predict the area it would affect, and the likelihood of future stroke. Also, a 3D MRI is synthesized that would aid doctors in making a more accurate and earlier diagnosis [48]. A convolutional network-based model was developed to ensure visibility of more distal arteries in the late dynamic phases, which was found to be of prime importance in diagnosis in people with decreased blood flow [49]. Shahid et al. (2024) [28] used a 3D CNN model to allow detection of CSVD at an early stage (stage 1), which is usually asymptomatic, to reduce the burden of morbidity and mortality associated with the same and reduce the risk of future complications [28].

A lot of these models did not provide a method for continued monitoring of the lesions, which is essential, considering the progressive nature of CSVD. An AI-assisted compressed sensing to detect and follow static lacunae lesions was found to be time efficient with better quality images for the follow-up on CSVD [50]. Another associated drawback was the differentiation of lesions that appear like the above findings. Few models have been developed that could assist with the same. Rashid et al. (2021) [51] developed a DL model for differentiating CMBs and iron bleeds in MRI. Kim et al. (2024) [52] used an automated magnetic resonance measurement to establish a differential diagnosis between CSF1R-related leukoencephalopathy and subcortical ischemic vascular dementia caused by CSVD based on WMHs thickness and cortical thinning [52].

Predicting the Development of CSVD and Disease Severity

Lambert et al. (2015) [18] found a distinct anatomical variant of cortical atrophy and thinning that can be equated with the disease severity, which was found to be noticeably different from that seen in ageing (more prominent changes bilaterally in the dorsolateral prefrontal, parietal, and posterosuperior temporal vortices; ageing: occipital and sensorimotor). The volume of WMHs in the MRI was postulated to be a quantitative measure of the disease severity, establishing an affiliation between the white and grey matter using a Gaussian process model regression, a probabilistic linear regression technique [18]. One study developed a linear regression and ML predictive models to establish the role of small vessel disease burden (measured using total CSVD score: lacunae, micro bleeds, basal ganglion EPVS, and WMHs (periventricular > deep) in predicting the functional outcome and risk of acute ischemic stroke (AIS), which brought out superior results when compared to prediction using regular clinical data (extreme gradient boost model was the most efficacious) [53].

Gibson et al. (2024) [54] developed a CNN to quantify WMHs and predict disease severity [54,55]. DS GAN is a DL model that was used to derive fractional anisotropy and mean diffusivity from MRI T1 imaging rather than from the more sensitive diffusion tracer imaging, to ensure quicker prediction of the dementia risk in CSVD. It was found to be a better predictor than WMH volume and produced results that were analogous to the ground truth [56].

A possible link between temporal variations in dynamic functional connectivity (DFC) and the development of CSVD was studied by Chen et al. (2024) [57], which included the analysis of alterations in mean dwell times, fractional windows, and transitions using a sliding window approach and k-means clustering. This opened up a new marker to predict CSVD. Further research in longitudinal extensions can help in predicting the progression of disease.

Zee et al. (2021) [58] conducted a study that emphasized an ML model to localize the WMHs based on MRI images of retinal vessels as an inexpensive and effective screening tool to predict the possible outcome and the probable symptomatology [58]. Another study conducted by Cho et al. (2022) [59] established fundus photographs as capable of partially predicting WMHs [59]. Yang et al. (2024) [60] came up with a combined risk stratification model, which included ML and MRI to predict stroke within three years in patients with CSVD [60].

AI-assisted models for gait analysis (Ready Go Motor Evaluation System) and eye tracking (Eye Know assessment system) have been postulated to be good screening tools for predicting the cognitive decline in patients with CSVD.

Eye tracking (low anti-saccade accuracy), gait (lower step speed, shorter length, longer stance time, longer swing, larger step width, gait asymmetry), slower bilateral stride, and left swing velocities (worse cognition) overcome the language barriers. However, we still need to develop systems that can combine both and extend domain-specific cognitive decline [61].

Zhu et al. (2022) [62] incorporated an intelligent algorithm to analyze electroencephalograms to study CVSD associated with cognitive impairment. Not only did this AI-based model detect the vascular cognitive impairment with no dementia (VCIND) and vascular dementia (VD), but it also predicted the progression of VCIND to dementia (background changes with abnormal waves with dementia and only background changes with no dementia) [62].

Despite being a newer area of research, various models have been proposed to assist in screening, prediction, diagnosis, and prognosis of CSVD. Also, establishing the possible etiology and complications of CSVD has also seen some contribution from the field of ML. Predicting the exact locations of the lesions has also helped in incorporating newer treatment modalities. Therapies like photobiomodulation (transcranial intranasal via oral cavity), which can target specific areas of the brain, can now be extended to the therapy of CSVD, which promotes self-repair of the brain by stimulating brain-derived neurotrophic factor (BDNF) synthesis and synaptogenesis [44]. Overall, it has helped in a more patient-specific diagnosis and therefore precise care, which ensured effective utilization of the resources and better outcomes. There are still a few areas where improvements can ensure even better outcomes in the future. First, a lot of the models involved had a smaller sample size or were restricted to a specific group (race, age, etc.), which made it difficult to establish the reliability of the results in the wider population. Second, there have been extensive efforts made to identify and quantify the imaging modalities; however, this needs to be further extended to follow up on these lesions, considering the nature of CSVD lesions to keep progressing. Third, a more holistic approach toward integration of imaging markers, clinical features, molecular and protein level studies, and ensure more specific and reliable results [43]. Fourth, more advanced AI models, including transfer learning and human-in-the-loop learning, can further ensure effectiveness. Wen et al. (2023) [63] established functional near-infrared spectroscopy and diffusion correlation spectroscopy to be a more holistic evaluation of cerebrovascular disease and associated psychiatric illness. Fifth, a lot of the latest information has come to notice regarding CSVD and DL models that can be extended for detecting these. Yan et al. (2024) [64] identified the thalamic covariance network as a prominent biomarker to detect early cognitive decline in patients with CSVD [64]. Raposo et al. (2021) [65] considered the peak width of skeletonized mean diffusivity as a marker for cognitive decline and structural disruption in CAA [65].

AI in the Diagnosis of Ischemic Stroke

One of the crucial aspects of ischemic stroke diagnosis is identifying the occlusion of the vessel that is responsible for the stroke, as it is crucial in determining the severity of the stroke and selecting appropriate treatment (e.g., thrombolysis and thrombectomy). So, one of the main applications of AI-based technologies is to detect LVO. Multiple AI technologies, such as Viz LVO, RAPID LVO, CINA, HALO, and Brainomix [17,66], have developed algorithms that can detect LVO from CT angiography (CTA). One of the first FDA-cleared CNN-based tools is Viz.ai, which reported an AUC of 0.91, sensitivity of 70%, specificity of 90%, accuracy of 90%, and a negative predictive value (NPV) of 79-99% for LVO detection on CTA [67-69]. RAPID LVO reported an NPV of 97-99%, sensitivity of 87%, and specificity of 94% [70,71]. Prehospital ML models for LVO triage demonstrate variable accuracy compared with the Rapid Arterial Occlusion Evaluation (RACE) scale, with prospective external validation remaining limited [11].

Another important aspect in the diagnosis of stroke is calculating the Alberta Stroke Program Early CT Score (ASPECTS) score. ASPECTS is a 10-point standardized scoring system on noncontrast CT that evaluates the middle cerebral artery (MCA) territory and plays a role in determining stroke severity, rapid triage, guidance regarding the type of treatment (thrombolysis vs. thrombectomy), and predicts clinical outcome (high chances of hemorrhagic transformation vs. less chances post tPA). Several AI-based software, such as RAPID ASPECTS [72,73] and Brainomix [74], can calculate the ASPECTS score. The ASPECTS score calculated by RAPID ASPECTS correlates well with neuroradiologist scoring [75-77].

In addition to the above factors, another critical imaging tool in the diagnosis of stroke is CT perfusion. CT perfusion quantifies ischemic core and penumbra, quantitative measurement of cerebral blood flow (CBF), cerebral blood volume (CBV), and mean transit time (MTT). These determine the decision regarding treatments (thrombolysis vs. thrombectomy). AI-based software such as RAPID-CTP, Vitrea CT, and FastStroke/CT perfusion 4D can generate perfusion maps, estimate infarct volume, and distinguish between salvageable and unsalvageable brain tissue. The AI-based tool RAPID-CTP shows a strong correlation with reference MRI reads, including diffusion-weighted imaging (DWI) and fluid-attenuated inversion recovery (FLAIR) [78,79]. A similar technology, i.e., FastStroke/CT perfusion 4D, can calculate infarct volumes and assess collateral perfusion [80,81].

AI in the Diagnosis of Hemorrhagic Stroke

Hemorrhagic stroke carries the burden of high mortality and morbidity with significant fatality rates. Multiple AI-based FDA-approved software are developed to diagnose ICH, such as BriefCase [82], RAPID-ICH, and Viz ICH [83]. Across studies, RAPID-ICH shows high NPV and variable positive predictive value (PPV), with sensitivity typically at ~92-96% and specificity at ~84-99.5%, depending on cohort, prevalence, and software version. These tools can identify the hemorrhage anatomical location, hematoma-expansion risk, and hematoma volume [84]. These algorithms support emergent ICH care by expediting detection and hematoma volumetry, enabling timelier neurosurgical planning and intervention. Notably, the evidence is mostly single-center and retrospective; PPV swings with prevalence, and false positives drive alert fatigue. Routine use should await multicenter, prospective trials demonstrating outcome gains, with external validation, calibration, and subgroup reporting.

Therapeutic Decision Support and Workflow Impact

AI tools help in prioritizing patients who require immediate treatment. Considering the narrow therapeutic window of stroke, early recognition is critical. However, identifying stroke in acute settings remains challenging, and any delay or missed opportunity for intervention not only increases healthcare costs but also significantly contributes to higher patient mortality [13].

With AI’s innovative potential, imaging interpretation is enhanced. Other than streamlining stroke diagnoses, AI is now capable of revascularization treatment outcome predictions, thus significantly improving patient selection for initial endovascular treatments. This reduces mortality and disability rates associated with acute ischemic stroke [6]. Several studies reported 18-25-minute door-to-needle reductions with AI-assisted triage [71,85,86].

There is still a shortage of AI tools in deciding patient eligibility for thrombectomy. Current research posits that AI can accurately derive extents of endovascular therapy eligibility through analyzing factors of clinical angiographic progress and complications following such treatments. However, AI’s long-term effectiveness in management and outcome forecasting remains limited. In a systematic review and meta-analysis of 16 studies evaluating 19 models to assess AI’s ability to predict 90-day functional, successful reperfusion, and hemorrhagic transformation, while ML and DL models have shown promise in accurately predicting post-treatment outcomes for LVO strokes, they are unable to consistently outperform traditional prognostic scoring systems. Findings show many models were developed using small datasets, lacking robust external validations and an elevated risk of bias. Hence, highlighting a necessity for further research into ML applications to enhance future decision-making [87].

Use of AI in early detection of complications like reperfusion injury/post-thrombolysis intracranial hemorrhage with the help of perfusion and diffusion imaging, and residual clot analysis, which might require additional thrombectomy. Prevalent algorithms of AI tools used in detecting complications post-thrombolysis, such as intracranial hemorrhage, include logistic regression (LR), support vector machine (SVM), and random forest (RF), with gradient boosting (GB). While having surpassed traditional scoring methods in their advanced versions, where clinical and imaging data have been combined, these algorithms have shown better predictive performance, a critical factor for decision-making. This reduces mortality and disability rates associated with acute ischemic stroke [6,88].

In telestroke, AI boosts diagnostic imaging analysis and interpretations through the application of ML algorithms, allowing the prediction of three-month prognoses and the course of patient recovery post stroke in hospitals. Additionally, AI models are being adopted into decision support systems to assess initial clinical presentations of patients and extract relevant clinical information from electronic systems [13]. AI can extract structured data from electronic health records (EHRs) to support decision-making; the real-world outcome benefit remains unproven. In other words, AI can provide crucial decision-making support and streamline care in addition to enhancing workflow and alleviating clinical care provider burnout during urgent critical cerebrovascular emergencies [8]. Most evidence is pre/post or single-center and confounded by concurrent workflow changes. Prospective multicenter trials with outcome endpoints and cost-effectiveness analyses are needed before routine adoption.

AI in Post-stroke Rehabilitation: Motor, Aphasia, Mental Health, and Cognitive Function

Natural recovery post stroke has always been subpar, rendering it necessary to provide immediate attention to the development of tools for post stroke rehabilitation. AI has shown promise to be an effective and secure option in post-stroke rehabilitation, and with the additional benefits of reduced manpower requirement, accessibility, and the ease of its application, it has proven to be more effective than the conventional methods over the majority of the scales [89].

AI in Rehabilitation of Motor Deficits

A lack of established criteria and procedures for ensuring precise outcome prediction, as per the kind of motor deficit, has paved the path for using AI-assisted technologies for pose and position estimation and custom hand grip measurement over the conventional methods for better uniformity and patient-specific outcomes [90]. Algorithms for real-time dynamic gesture recognition and models to assess the degree of bend and muscle strength in the patient, which then assist in completing the motion and providing appropriate support, have been tried out and found to be effective; however, certain safety concerns still exist regarding the extent of control over the robotic arm and the cumbersome equipment that patients have to wear. Further research is to ensure analysis of more complex variables and develop superior models to completely reproduce a human’s approach, and then be upgraded to ensure more appropriate, safe, and practical solutions as necessary. Evidence of AI-based algorithms’ ability to assess the results of the rehabilitation and ensure a more standardized approach remains preliminary. Utilizing a deep convolutional generative adversarial network model to generate artificial electroencephalography (EEG) data with established validity to overcome the scarcity of EEG data has shown promising results in bolstering the rehabilitation technologies, like the motor imagery brain computer interface [91]. Using DL techniques to analyze the complex data from wearable devices can ensure more reliable and superior activity recognition for better rehabilitation measures [92]. Abnormal neural oscillatory patterns during post-stroke periods can be assessed by BrainQ, an ML-based model that uses motor-related spectral features from EEG, magnetoencephalography (MEG), and electromyography (EMG). It uses low-frequency electromagnetic fields to alter the abnormal oscillations, which have proven to be more effective and safer than the traditional methods. Another model is by IpsiHand Upper Extremity Rehabilitation System, which leverages the use of an EEG electrode headset to identify neural activity of the intent to move from the undamaged areas of the brain and then utilizes that to induce motor activity. More extensive research on the same can ensure an at-home rehabilitation service post stroke, ensuring greater feasibility and functionality, and therefore ease the tedious process [66]. When compared with passive robot hand training, AI and EMG-integrated robot hand-assisted training produced superior outcomes with respect to the upper extremity (UE) motor function, amount of use, and spasticity [93].

Enabling the involvement of people with disabilities in recreational activities can ensure better recovery and outcomes and enhance the feeling of normalcy. One such attempt involved the use of AI-based technology in video games, which used adaptable algorithms to provide a full range of motion when the gamer is unable to do so, adjusting the difficulty level as per the performance, rendering them winnable and engaging and also provide appropriate rewards in terms of applause or messages after a win for a sense of success to promote the mental well-being of the patient. These models still need to be worked on by evaluating their efficacy in patients over 60 years old and developing more advanced algorithms to detect even minimal voluntary movements [94].

AI in Aphasia

AI has proven to be of extreme help in diagnosing the type of aphasia, along with assessment of severity and rehabilitation. A combination of speech signal processing, speech recognition and transcription, and speech analysis has been used in AI-assisted models to streamline and simplify the tedious process. Speech signal processing using CNN and recurrent neural networks helped in detecting non-fluent and fluent aphasia, respectively. To overcome the language barrier, attempts to convert speech spectrograms into time-frequency images, which can serve as training data for ML models, have also been conducted. DL-assisted speech recognition models depicted an accuracy of 98.1% in diagnosing aphasia as opposed to the classical software, which had an accuracy rate of just 70%. Integrating the clinical data with imaging analysis and EEG data has helped with classifying and identifying subtler forms of aphasia with more accuracy and precision. AI models have proven to be more effective in the treatment and rehabilitation of aphasia-affected patients because of their dynamic nature and the capability to readily evaluate and attain necessary feedback (self-directed rehabilitation training) to ensure a more precise treatment and rehabilitation plan. Virtual therapists (e.g., virtual therapists for aphasia treatment and oral reading for language in aphasia) use ML models to simulate real-life experiences and guide the patients through the exercises, which have shown at par and at times more positive outcomes when compared to those of human therapists. These are not without their limitations. More precise models need to be developed for a more fine-grained understanding of language. They fall short in addressing the emotional and social aspects of the treatment, so a combination of human and AI models would result in superior outcomes [95].

AI in Detecting Signs of Post-stroke Adverse Mental Outcomes

About 30% of post-stroke people end up with depression, with another 20% ending up with anxiety. A higher mortality rate and subpar recovery have also been observed in these patients. Oei et al. (2023) developed an ML model to predict the risk of these adverse mental outcomes, which produced an accuracy of 74%. This facilitates early intervention and prevention [96]. AI-based models can be developed to predict the probability of patients’ suicidal tendencies in post-stroke care based on psychological features and to appropriately classify them into high and low risk to ensure earlier intervention and better prevention strategies [97].

Longitudinal studies on the same can further support the reliability of these models when incorporated into real-life settings [96]. The models can further include the imaging and clinical aspects of stroke to ensure a more complete prediction of suicidal tendencies [97].

AI in Cognitive Rehabilitation

The cognitive functions can be judged based on four elements: judgment, memory, attention span, and quick responsiveness. Interactive games have been developed, aided with AI technology, to develop a user-friendly interface, using recurrent neural networks (RNN) to develop AI models to adjust the difficulty levels to provide an interactive experience, which showed better improvements in the cognitive function, more motivation on the part of the participants, and thereby better outcomes [98].

NeuroAIreh@b is one such ML-based program that coordinates the field of neurophysiology with clinical assessment to develop feasible, beneficial, and adaptable means of cognitive neurorehabilitation. This was tried out in a set of post patients with stroke, and promising results were attained. However, the validity and the reliability of the program need to be assessed by a randomized control trial, and the dataset used for training was that of Alzheimer’s dementia, which varies from the cognitive abnormalities post stroke, which also needs to be taken into consideration. The addition of virtual reality (VR) models to the same can ensure a more personalized experience [99].

Despite the promising results of AI in neurorehabilitation, it is of utmost importance that we realize that these models can supplement but not replace human discretion and expertise, and the ethical considerations regarding data privacy and patient autonomy must also be considered to ensure safe, beneficial, and responsible use of AI in healthcare [100].

AI in Remote Healthcare for Stroke

Telemedicine and remote wearables are of utmost importance to ensure that healthcare is accessible in the most remote of locations, especially in areas with low physician-to-patient ratios. A pandemic situation like COVID-19 has further emphasized the need for these facilities to ensure continued care for the patients [101,102]. This becomes even more valuable in diseases like stroke, wherein appropriate diagnosis and early intervention, and the prolonged requirement for follow-up, are of prime importance. Combining AI models with telestroke is something that can ease the process for both the patients and clinicians by ensuring earlier, quicker, and more accurate diagnosis, reducing delays, ensuring organized workflow, and reducing the burden on the healthcare provider [8]. Large language model-based ML programs can overcome the language barriers and ensure a more effective diagnosis at the dispatcher level, analyze the health data by incorporating AI in emergency medical services systems, along with real-life global positioning system (GPS) data of emergency vehicles. Image-based DL models in camera-equipped ambulances can ensure early identification of stroke [103]. Real-time monitoring of risk factors in telemedicine facilities using AI for predicting stroke, and close monitoring of physical activity, blood pressure, and heart health to prevent complications and ensure better recovery is noted. This approach ensures timely and personalized risk assessment of stroke, thereby allowing interventions to be tailored accordingly [102]. Using AI-based models to track movements from simple videos can ensure more widespread motor assessment and advanced tele-rehabilitation strategies [104].

AI-assisted wearable devices can ensure more continuous and real-time tracking of various risk factors of stroke and provide better prediction of the same, in contrast to the traditional methods of screening, which involve regular hospital visits. Currently, wearable devices are in use for continuous ECG and blood pressure monitoring, recording heart rate and physical activity, and detecting atrial fibrillation and sleep patterns. Wearable sensors ensure a real-time feedback loop to develop personalized rehabilitation plans and adaptable models as per the requirements and progress of the patient [102].

A hydrogel-based optical waveguide stretchable (HOWS) sensor, which integrated sensing, wireless transmission, and AI for monitoring the hand and joint movement, was found to be effective in early diagnosis and rehabilitation monitoring in patients with stroke [105]. Chae et al. came up with a web-based upper limb home rehabilitation system using ML and smartwatch models, which showed better functional recovery in the Wolf Motor Function test and ROM of flexion and internal rotation in chronic stroke survivors with an accuracy of 86.5% to 100% along with a decreased dropout rate and better mental outcome [101].

The convenience and the limitless potential of these devices make them attractive options, but they are not devoid of drawbacks. The reliability of the data is still under question, as the data obtained are highly dependent upon the user's compliance with the instructions, which need not always be accurate, and so is the variability of the sensors used in various wearables. Overcoming this would require coherence and more standardized guidelines in terms of findability, accessibility, interoperability, and reuse (FAIR) of the devices that must be developed interoperability, which is integrating the data from other wearables with other clinical data available, which can further enhance the viability. Extending and ensuring the use of these devices to all groups of people in terms of race, gender, social standing, etc., is important to ensure a more complete dataset for drawing conclusions [106].

The privacy of this data is also under question. Increased dependence on these devices has led to decreased healthcare checkups and other traditional practices. It needs to be understood that these devices must just supplement and not replace more traditional healthcare practices. The expensive nature of these devices has shed some light on the accessibility of these devices and the equity in healthcare. Making these devices available to all and ensuring greater access to the data and the interpretation tools can ensure greater transparency and trust in the feasibility of these devices among the masses [106].

The limited battery life and power consumption are also a matter of concern. More energy-friendly models need to be developed [107].

Application of AI in Preventive Medicine of Stroke

Preventive medicine plays a key role in stroke prevention. Preventive medicine in stroke involves understanding, identifying risk factors, risk stratification, early detection, and implementing targeted preventive strategies. Risk factors of stroke can be categorized into non-modifiable risk factors, such as genetics, and modifiable risk factors, such as hypertension, diabetes mellitus, and atrial fibrillation. By recognizing risk factors, individuals at high risk can take preventive measures to reduce the likelihood of stroke. AI transforms stroke prevention by enhancing and detecting risk factors, risk stratification, generating AI-based predictive models, and tailoring strategies accordingly for prevention [108].

Role of AI in Non-modifiable Risk Factors

The standard risk factors of stroke are age, total cholesterol, low-density lipoprotein, hypertension, diabetes mellitus, and many other factors. Yet, they cannot efficiently foresee events like a stroke in the future [108]. Hence, other biomarkers, such as genetic variations, have been identified to provide insights into stroke pathogenesis. Primarily, two types of genetic variations, i.e., monogenetic and polygenetic variations, can cause stroke. Monogenetic (Mendelian inheritance) is less common. A few common examples of monogenetic abrasions that result in stroke are CADASIL (NOTCH-3 gene) [109] and CARASIL (HTRA1) [110]. Most of the strokes are polygenetic with multiple genetic variations. Hence, GWAS is conducted to identify the genetic variations and the calculated PRS [111]. PRS has the potential to predict stroke and risk stratification of stroke as well [112].

AI-based algorithms can generate PRS scores better than traditional methods [113]. Traditional PRS-generating methods are more linear, and AI-based PRS algorithms are nonlinear (reducing the dimensionality of extensive genomic datasets) and make it more precise and accurate. Also, the ability of AI-based algorithms to handle vast datasets from biobanks, GWAS, and multiomics data and to integrate multiomics data (genomic, transcriptomic, epigenomic) improves the scalability and efficiency of PRS calculations [114]. As a result, AI-based PRS calculators have the potential to accurately predict stroke in comparison with traditional methods [115].

Some modifiable risk factors of stroke include hypertension, diabetes mellitus, cholesterol, and atrial fibrillation, which result in blood vessel damage, atherosclerosis, and stroke. Early detection and continuous monitoring of these risk factors can reduce stroke incidence through timely intervention. AI-based models can continuously monitor and gather real-time data, which can detect these modifiable risk factors early and reduce the incidence of stroke. They can also give predictive analysis about stroke and identify high-risk individuals for proactive interventions. An important modifiable risk factor in stroke pathogenesis is hypertension. Therefore, significant advancements in AI have led to the innovation of various wearable devices, such as smart watches, wrist bands, finger monitors, and skin-compatible sensors. These devices are integrated with AI-based models, which work with the principles of the photoplethysmography method [116]. These smart devices are integrated with the smartphone. There are multiple benefits of these smart devices, such as continuous real-time monitoring of blood pressure, which can be saved for long-term tracking and shared with physicians [117], and alerts the user if the blood pressure is above the physiological values, which gives an opportunity to get an early intervention if required.

Another important risk factor for stroke is diabetes mellitus. Large glucose fluctuations can impact stroke outcomes. Hyperglycemia worsens stroke outcomes such as increased infarct size, blood-brain barrier disruption, and higher chances of hemorrhagic transformation [118]. Therefore, continuous glucose monitoring allows early detection and treatment of hyperglycemia by adjusting insulin. Most devices can continuously monitor glucose by either invasive methods, such as placement of a subcutaneous sensor, or noninvasive methods, such as infrared rays/spectroscopy [119]. Integrating AI in continuous glucose monitoring (CGM) systems gives continuous, real-time data, which can be shared with physicians. It can also help in timely interventions if required, such as altering insulin dose and eventually improving patient outcomes [120].

Another significant risk factor is atrial fibrillation, which is responsible for cardioembolic stroke. Early detection of atrial fibrillation can be achieved with continuous ECG monitoring, which can detect irregular heart rhythms. AI-based models can detect atrial fibrillation either by photoplethysmography (PPG) or single-lead ECG [121]. In comparison with traditional methods, it can monitor an extended amount of time, which increases the chance of detecting abnormal rhythm [122]. Another study on the use of AI models in predicting abnormal ECG in atrial fibrillation showed excellent results with a recall of 81%, specificity of 78%, F1 score of 75%, and overall accuracy of 78% [123].

AI-based models can analyze complex datasets and identify age and modifiable and non-modifiable risk factors, and give a score that can predict stroke risk. This helps to mitigate the prevention strategies. PRERISK is a predictive model developed based on ML and can predict stroke recurrence. The AUC of early recurrence was 0.76, late recurrence was 0.6, and long-term recurrence was 0.71 [124].

Technical and Practical Considerations in Clinical Deployment of AI in Stroke

The preference of CNN over traditional ML methods has demonstrated various advantages in stroke diagnosis through imaging. CNN can identify subtle findings and intricate details of stroke patterns from the imaging modalities like CT and MRI, which eliminates the need for manual intervention, which most of the ML models rely on. Also, the ability of CNNs to detect hierarchical patterns (lines, edges, textures, tumors) in complex datasets has been a breakthrough in medical imaging analysis [125,126].

The CNN can manage complex 2D/3D data and segment ischemic lesions in stroke in comparison with traditional ML models, which are essential for planning the treatment [127]. Additionally, CNN can localize the location of the lesion (spatial hierarchy) and spatial relationships, which are also crucial to predict stroke outcomes [128]. The sensitivity of CNN in predicting stroke is 93-96.5% while ML models like support vector regression (SVR) can never reach that level of accuracy, as they cannot manage complex tasks [129].

CNNs are versatile and adaptable, and can learn from large pre-trained models, whereas ML models are not adaptable and cannot leverage pre-trained datasets, which require building the model from the beginning [130]. Although CNNs might require large datasets, which can be challenging, they outperform traditional ML models in handling complex datasets, in identifying structural and spatial hierarchical patterns, and in providing accurate stroke predictions across various clinical scenarios of stroke.

Transfer Learning

Transfer learning is a method in which an AI model is chosen that is already trained in large datasets (medical/non-medical) and fine-tuned to a different task, such as stroke diagnosis. In 2018, Chilamkurthy et al. used pre-trained CNN models, such as the ResNet model, which was fine-tuned to detect intracranial hemorrhages on CT scans [131]. Transfer learning in stroke diagnosis leverages fast learning and has less computational cost instead of training the models from scratch, although a key challenge can be a domain shift.

Model Interpretability

When a particular AI model predicts a stroke, and there is no explanation, it is called a "BLACK BOX" issue. This leads to a lack of trust and accountability. Explaining the ability of the AI model is extremely important for clinicians to trust and justify the decision made by it. The more transparent AI models are about their decision-making, the more clinicians will trust the model and adopt it in clinical practice [132]. AI can clearly demonstrate the decision-making process and can ensure adherence to regulatory standards [133]. Explainable AI helps in identifying errors and the reasons for errors, which can help in improving the models [132].

Various methods are used in generating saliency maps and heat maps, such as GRAD-CAM, guided propagation, SHAP, and LIME. These maps highlight the regions responsible for the decision-making process. Grad-CAM and back propagation are visualization techniques [134], which are specifically used in CNN-based models by highlighting the areas for decision-making, whereas SHAP and LIME explain which pixels or image features played a role in the prediction of stroke of any model [135,136].

Even if it is explainable, AI techniques can be computationally intensive. It is important to enhance trust among clinicians, to comply with regulations, and to enhance patient safety.

AI Model Calibration

AI model calibration is a process where the raw data output given by ML models is adjusted by techniques, such as Platt scaling and isotonic regression. This process is done to ensure the output reflects the true clinical scenario instead of overestimating/underestimating the predictability of stroke. Platt scaling uses logistic regression, which is a parametric calibration technique that assumes the sigmoid relationship for calibration and predominantly uses ML models like SVM [137]. Isotonic regression is more flexible and uses non-parametric calibration techniques and shows superior performance compared with Platt scaling [125].

Calibrated models are more dependable in stroke prediction and hence more useful clinically in decision-making. The calibrated models perform better in stroke prediction, which prevents the need to retrain the models [138]. Calibrated AI models for stroke prediction significantly enhance stroke diagnosis, thereby improving patient outcomes.

Integration of AI Into Real-Time Clinical Workflows

Multiple AI-driven solutions such as RapidAI, Brainomix, and Viz.ai have been developed and integrated into real-time clinical workflows for the assessment of stroke [67]. These tools assist in intracranial hemorrhage detection, LVO detection, ischemic core and penumbra assessment, and ASPECTS scoring. Although primarily designed for acute stroke rather than CSVD, they serve as implementation benchmarks, illustrating regulatory clearance, picture archiving and communication system (PACS)/workflow integration, and post-deployment monitoring, which are directly informative for CSVD-focused AI applications.

Integration of AI models into the clinical workflow is a critical aspect of successful deployment. These AI tools, which are developed for stroke diagnoses, are first integrated with PACS, so whenever a brain scan is done for stroke, the images are automatically pushed into the AI system. The analysis of the scan is done by the AI system in parallel to the routine care. Once the analysis is done, pop-up alerts appear on the workstations of both the treating neurologists and radiologists. Radiologists and neurologists can view the AI-generated report and hasten the decision-making even before a formal report is issued [139]. Despite the promising role of AI in stroke diagnosis, there are many challenges in adopting it in real-life clinical scenarios.

Technical Challenges

Integrating an AI system in clinical workflow requires a compatible PACS system, high-speed internet, DICOM compatibility, and vigilant data security measures. Also, the large DICOM files would be transferred without any time delay from PACS to the AI server or cloud-based processing. If it is not optimized, the latency over a few minutes can nullify the benefits of AI in stroke diagnosis [136].

Regulatory Challenges

The clearance of AI models has been for static versions, but there is no clear mechanism on how to regulate continuously learning models. A post-market surveillance of AI models is required, which requires collaboration between AI vendors, users, and regulators, which can be challenging.

Ethical Challenges

Real-world deployment requires clear accountability, explainability, bias audits, and calibrated outputs suited to local prevalence and imaging protocols. Privacy-preserving training (e.g., federated learning) and post-market monitoring can reduce risk while maintaining clinical performance.

Financial Challenges

Integration of AI in clinical workflow is expensive to maintain the systems, such as cloud-based systems, requiring a supportive team, cybersecurity tools, etc., which can be a financial challenge for the hospitals.

There are quite a handful of challenges present to integrate AI tools into clinical workflow; the positive outcomes the tools bring about in stroke workflow, such as detection of hemorrhages and ischemia in minutes, and accelerating treatment pathways, outweigh the challenges. If the challenges are managed, AI has the capacity to transform acute stroke management, improving patient outcomes.

Limitations of AI in Stroke

ML and DL models require high volumes of sample data for them to perform with high precision and limited errors [140]. A large amount of data is also essential for the models to be validated. Unavailability of a large volume of standardized datasets with high-quality images, high-quality clinical information, and follow-up data is a challenge in applications of AI in stroke. Many public datasets should be made available. Public data sharing challenges can cause a breach in data sharing and compromise privacy protection. To overcome this, a novel ML method called federated learning is being developed, wherein it sets up a global model with a central server and distributes it to all participating devices. This model also ensures less data leakage and good data privacy protection by limiting the data in the local device and sending it back to the central server without transmitting the actual data [141-144].

Interpretability, Transparency, and Challenges of AI Models

Many ML models function as “BLACK BOX”. They generate predictions without showing how input features drive those outputs, reducing transparency and clinician trust in AI-based decision-making [145].

Ethically, deployment should ensure informed consent, privacy-by-design (e.g., de-identified storage and role-based access), transparency about model scope/limits, a clinician override, subgroup fairness checks (e.g., older adults/diabetes), and post-deployment monitoring (e.g., drift alerts and audit logs), especially when using home/wearable data.

Adopting explainable techniques (e.g., saliency/Grad-CAM heatmaps for CTA/non-contrast CT and SHAP for clinical/tabular models) and calibrated probabilities with pre-specified decision thresholds, together with versioning and audit trails, prospectively evaluate whether explanations actually improve clinical decisions. How to overcome the limitation: development of explainable ML algorithms in the stroke workflow, which explains the performance of the algorithms and incorporates trust among the clinicians to adopt them in the clinical workflow.

Challenges to Incorporating AI in the Clinical Workflow

Real-world stroke diagnosis often relies on the integration of diverse data sources, such as a combination of patient history in EHRs, neuroimaging in PACS, radiology information system (RIS), clinical biomarkers in laboratory data, and genetic profiles. Many existing AI models are limited by their reliance on a single data type, such as MRI or CT scans. These make comprehensive assessments of a presentation of cerebrovascular disease difficult and cause disruptions to already existing routine practices [146]. To overcome the limitation, developing robust algorithms with high predictive performance, and close attention has to be given to how to integrate the algorithms in the existing clinical workflow seamlessly, without interrupting the existing system.

Due to the black box nature of AI algorithms, the rationale behind the predictions and the suggestions made by the algorithm remains foggy, as well as the lack of familiarity with the technological aspects of the same, because of which the reliability and the trust of the clinicians over these algorithms remain debatable. Also, the legal implications of a deleterious outcome due to an algorithm prediction, which usually still falls on the physician in charge, further make it difficult to depend on this algorithm entirely [147].

Incorporation of “Applied AI” topics in the medical student curriculum and the use of AI-driven devices to deliver clinical knowledge are being researched. Continuous training programs, including conferences and hands-on workshops, can instill a sense of trust in the AI systems among the clinicians and ensure seamless integration of this technology in the clinical decision-making system [147]. This training, given in line with the implementation of the technology, was found to be useful. At times, patient empowerment regarding self-care and remote monitoring devices must also be done. However, the time factor in an already hectic profession still needs to be addressed with user-friendly and time-saving interfaces [148]. The inherent bias in AI algorithms, per se, in the training data or the basic design of the algorithm can lead to fallacious outcomes. Assiduous data collection and regular data updating, auditing the algorithm, and developing ML algorithms that are “fairness aware” can help overcome this obstacle [147].

The clinicians can consider these systems a threat to their autonomy, and inherent in it is the resistance to change [147]. Also, there is the added fear that extensive dependence on these AI-based predictions can reduce the volume and depth of knowledge the future doctors possess [148]. Understanding the specific requirements of the clinician and the settings around him and developing a tailored interface can ensure a better integration of these technologies into the clinical settings [147].

Standardized data formats, seamless interoperability, and strong and appropriate data governance frameworks are necessary for the implementation of AI into the EHRs, medical devices, and clinical settings [147]. To ensure this, a collective and combined effort on the part of the clinicians, technology experts (data scientists and engineers), and the governing bodies is required to develop algorithms and systems best suited for the clinical setting in picture [147]. In addition, there is a necessity for a quantitative assessment of the effects (clinical and organizational) and the qualitative assessment of the experience of the clinicians and patients incorporated with its evaluation and valuation to ensure better feedback and improvements. The future must also be kept in mind, and continuous and zealous attempts have to be made to adapt these systems to changing times [148].

Data Bias Concerns

The performance of the ML algorithms is heavily based on the quality and diversity of datasets. Hence, if the datasets lack a robust inclusion of a diverse sample of population demographics, balanced cases, low-quality data, and non-standardized annotations, the model’s accuracy and reliability will suffer.

One of the major challenges in current AI-driven stroke diagnosis is the inherent class imbalance in existing AI training datasets. Since ischemic strokes are more common than hemorrhagic strokes, ischemic strokes are disproportionately overrepresented compared to other strokes. Consequently, this imbalance negatively impacts the ML algorithms, predictive accuracy, thus increasing false-negative rates for underrepresented stroke types. Training sets require balanced sample distributions.

High-quality training data are paramount to improve and ensure consistent accuracy of DL algorithms. The accuracy of AI models is also heavily influenced by the quality of annotations provided for training data, particularly in medical imaging. Fundamentally, three dimensions are considered in the construction of the training data. Firstly, the training setting for the algorithm, when raw CT or MRI is used for learning. Secondly, the validation set for the algorithm's hyperparameter tuning, where the best parameters are used to fine-tune models. Lastly, the testing set for algorithm performance evaluation, i.e., the usage of completely unseen data to evaluate the AI model’s final performance. A good ratio, for example, a recommended ratio of 70%, 10%, and 20% for training, validation, and testing, respectively, is needed, or there would be an overlap at patient level (a repetition and data leakage across training and test sets), which consequently generates a false high performance accuracy during testing, but poor performance in real-world clinical settings and new patients. The sources of the acquisition of these data, division methods, and accordance, as well as data allocation ratio, are also paramount.

Automated and AI-assisted validation tools of the annotation process are increasingly deployed. These include AI-powered second readers, hybrid annotation (AI suggestion with expert correction), and automated consistency checks (triggered alarms for second reviews by human annotators in case of AI-reported inconsistencies). Current annotations, however, can be assisted by ML tools. Today, the gold standard for medical image labeling is manual annotation by experienced radiologists.

To enhance the robustness of AI model training, datasets should be large and enriched with a broader spectrum of cases. Proper documentation and standardized coding, such as using the International Classification of Diseases (ICD-10), is crucial for ensuring consistency in data preprocessing and model training. The dataset for training should also include annotations from expert radiologists, according to standardized protocols, to assist in developing the model’s predictive accuracy and ability to identify complex patterns and relationships.

Cost-Effectiveness of Algorithms

Integrating AI algorithms into the clinical workflow of stroke can be expensive as it requires huge licensing fees, technical support expenses, continuous model update charges, the expense related to integration of the algorithm into the workflow, and training expenses [149]. By adopting open-source AI models, which can reduce the licensing fees, or a provision in which public and private institutes collaborate, the expenses can be shared. More research should be done on developing AI algorithms that are robust yet flexible, interoperable across various workflows, and easier to optimize, which can help in mitigating the costs.

Regulatory Concerns

AI-based stroke algorithms face challenges related to ethical and legal concerns, as the AI tools fail to manage legal risks and accountability. Development of robust AI algorithms and a blueprint of policies should be implemented. This requires collaboration of healthcare professionals, policymakers, and AI developers to develop a framework that can create a more ethically and legally compliant and transparent system [149].

Future directions and recommendations

Multimodal Data and Feature Engineering

Develop fusion DL models that combine neuroimaging with clinical/EHR and genetic data. Use feature-extraction pipelines for both structured fields (labs, medications, diagnoses) and unstructured notes via natural language processing (NLP), e.g., derive vital-sign trends and extract symptoms/diagnoses from reports to build comprehensive patient profiles. Evaluate workflows that integrate imaging features with blood biomarkers (e.g., CRP, interleukins, and endothelial dysfunction markers) for earlier risk stratification.

Data Quality and Labeling

Mitigate class imbalance with SMOTE (Synthetic Minority Over-sampling Technique), judicious undersampling, and class-balanced/focal loss. Ensure proportional coverage of ischemic/hemorrhagic phenotypes and severity strata and broad population diversity (age, sex, ethnicity, comorbidity, geography, care settings). Use standardized annotation with adjudication and report inter-rater reliability (Cohen’s κ-weighted for ordinal scales and intraclass correlation coefficient (ICC) (2,1) for continuous measures).

Study Design and Reporting

Train/evaluate with patient-level, leakage-free splits (ideally, temporal or site-held-out). Require external, multi-site validation; report calibration (reliability plots/Brier) and decision-curve/net-benefit with pre-specified thresholds tied to the task (e.g., LVO triage, ICH detection, and WMH/EPVS/CMB quantification). Provide subgroup performance (older adults, diabetes, multi-ethnic cohorts).

Privacy-Preserving Collaboration

Use federated/secure multi-site learning and stress-test domain shift across scanners and protocols.

Deployment and Governance

Implement bias/fairness audits, post-deployment drift monitoring, versioning, and audit trails, and maintain a clinician-in-the-loop with override. Assess workflow fit (PACS/EHR) and cost-effectiveness alongside clinical endpoints to justify adoption.

Limitations

This is a narrative synthesis based on a single database (PubMed) and English-only publications (2015-2025). We did not perform a PRISMA-guided meta-analysis because of substantial heterogeneity in study designs, populations, imaging protocols, reference standards, and outcome definitions; therefore, cross-study performance comparisons should be interpreted with caution. Although we used predefined inclusion/exclusion criteria, dual independent screening, and multi-reviewer adjudication in Rayyan, selection and publication bias may persist, and grey literature or non-indexed reports were not systematically included. Reported metrics occasionally come from vendor-developed tools, which may introduce reporting bias. Finally, the field is evolving rapidly; newer studies released after our search window may not be captured.

## Conclusions

AI now spans stroke prevention, diagnosis, treatment selection, and rehabilitation, with growing utility for CSVD (e.g., automated WMH/EPVS/CMB quantification and risk stratification). While evidence supports gains in triage speed and diagnostic consistency, broad clinical adoption requires high-quality, standardized data, external and calibrated validation, clear governance (accountability, safety, privacy), and cost-conscious integration into workflows. With these safeguards, AI can contribute to better, more timely stroke care.
